# Ammonificins C and D, Hydroxyethylamine Chromene Derivatives from a Cultured Marine Hydrothermal Vent Bacterium, *Thermovibrio ammonificans*

**DOI:** 10.3390/md10102300

**Published:** 2012-10-19

**Authors:** Eric H. Andrianasolo, Liti Haramaty, Richard Rosario-Passapera, Costantino Vetriani, Paul Falkowski, Eileen White, Richard Lutz

**Affiliations:** 1 Center for Marine Biotechnology, Institute of Marine and Coastal Sciences, Rutgers, The State University of New Jersey, New Brunswick, NJ 08901, USA; Email: andriane@marine.rutgers.edu (E.H.A.); haramaty@marine.rutgers.edu (L.H.); rrpassapera@gmail.com (R.R.-P.); vetriani@marine.rutgers.edu (C.V.); falko@marine.rutgers.edu (P.F.); 2 Center for Advanced Biotechnology and Medicine, Department of Molecular Biology and Biochemistry, Rutgers, The State University of New Jersey, 679 Hoes Lane, Piscataway, NJ 08854, USA; Email: whiteei@umdnj.edu

**Keywords:** marine natural product, deep-sea hydrothermal vent, drug discovery, induction of apoptosis, bacteria, computational methods

## Abstract

Chemical and biological investigation of the cultured marine hydrothermal vent bacterium, *Thermovibrio ammonifican* led to the isolation of two hydroxyethylamine chromene derivatives, ammonificins C and D. Their structures were elucidated using combination of NMR and mass spectrometry. Absolute stereochemistry was ascertained by comparison of experimental and calculated CD spectra. Biological evaluation and assessment were determined using the patented ApopScreen cell-based screen for apoptosis-induction. Ammonificins C and D induce apoptosis in micromolar concentrations. To our knowledge, this finding is the first report of chemical compounds that induce apoptosis from the cultured deep-sea marine organism, hydrothermal vent bacterium, *Thermovibrio ammonificans*.

## 1. Introduction

Deep-sea hydrothermal vents embed a large variety of organisms which are believed to display different types of metabolisms based on the comparison of their growth rates and chemosynthesis to their counterparts from shallow water [1,2]. These large and almost untapped reserves of organisms from the deep sea are being investigated as potential natural product sources [2].

With the lack of effective agents to control a spectrum of deadly cancers and viruses (e.g., HIV) and with drug-resistant microbes reaching epidemic proportions, pharmaceutical firms are actively searching for novel biodiversity to screen for bioactive natural products. In the search for sources of new chemical diversity, deep-sea natural products have emerged as a new potential and hot spot for drug discovery and development [2]. Life in the deep sea involves exposure to extremely harsh environment conditions (high pressures, variable temperatures and low oxygen and light) requiring its inhabitants to adapt their biochemical machinery to cope with these extreme conditions. This has probably the potential to affect both their primary and secondary metabolic pathways [3], giving rise to structurally unusual and unique metabolites.

**Figure 1:**
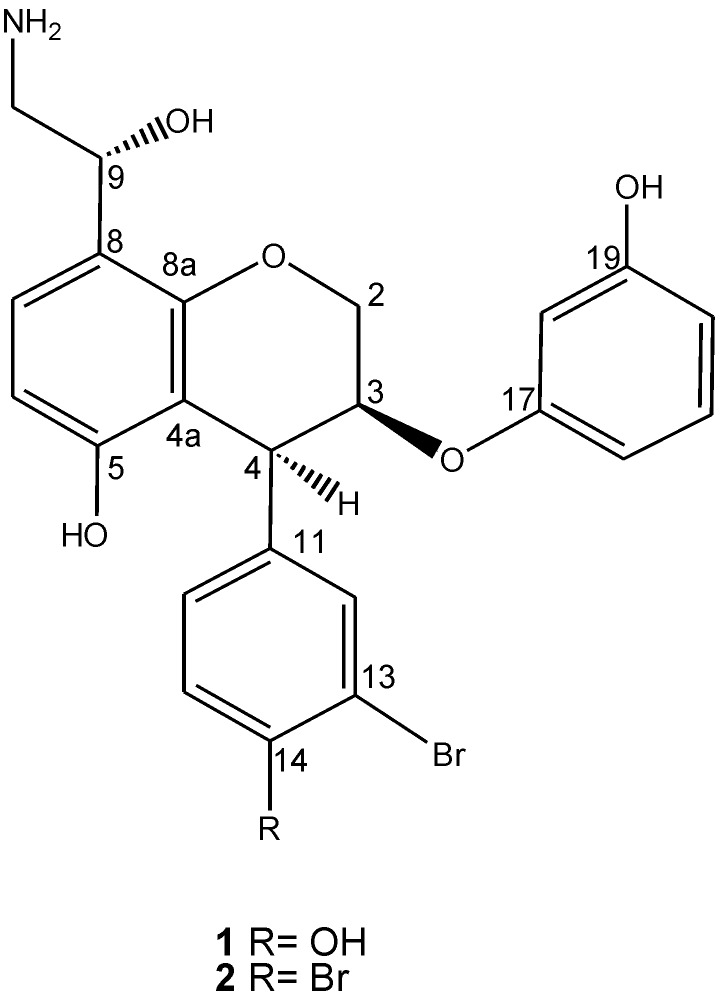
Structures of ammonificins A (**1)** and B (**2**).

Previously, we isolated ammonificins A (**1**) and B (**2**) (Figure 1) from a cultured deep-sea marine organism *Thermovibrio ammonificans*. The structural feature of **1** and **2** is unique and unprecedented, since they are never seen or isolated from shallow-water organisms, with the co-occurrence of hydroxyethylamine and phenol or brominated phenol with chroman. Albeit interesting, these compounds are practically inactive in apoptosis induction assay. In our ongoing effort to isolate bioactive compounds from deep-sea marine organisms and particularly to address the issue of which compound is responsible for the activity previously observed from the extract of cultured *Thermovibrio ammonificans*, we reinvestigate the ability of *Thermovibrio ammonificans*, a chemolithoautotrophic bacteria, to produce novel secondary metabolites. 

## 2. Results and Discussion

After careful analyses of the HPLC trace from our previous work, few minor peaks are considered for the object of the re-isolation. Using the same method of culture and strategy of fractionation and isolation in order to reproduce and re-isolate not only **1** and **2**, but also the other minor peaks for reasonable material for NMR and bioassay, 40 g wet weight of cultured organism was extracted in MeOH. The organism, *Thermovibrio ammonificans*, a thermophilic, anaerobic, chemolithoautotrophic bacterium, was isolated from the walls of an active deep-sea hydrothermal vent chimney on the East Pacific Rise at 9°50′ N, depth 2500 m in November 1999 and April 2000. Cells of the organism were Gram-negative, motile rods that were about 1.0 μm in length and 0.6 μm in width. Growth occurred between 60 °C and 80 °C (optimum at 75 °C), 0.5 and 4.5% (w/v) NaCl (optimum at 2%) and pH 5 and 7 (optimum at 5.5). The generation time under optimal conditions was 1.57 h. Growth occurred under chemolithoautotrophic conditions in the presence of H_2_ and CO_2_, with nitrate or sulfur as the electron acceptor and with concomitant formation of ammonium or hydrogen sulfide, respectively [4].

One part of the extract obtained from the MeOH soluble extract was dissolved in DMSO and retested for apoptosis induction assay [4,5,6,7] as verification. This assay is used to identify compounds that possess proapoptotic, and potentially anticancer, activity. 

**Figure 2:**
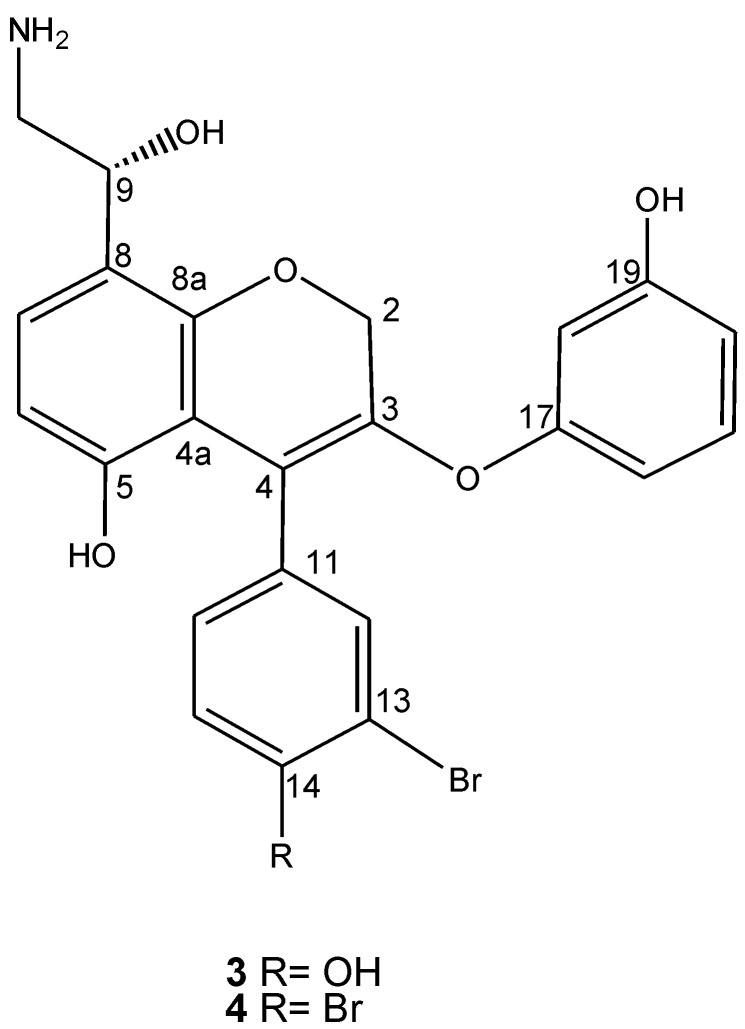
Structures of ammonificins C (**3)** and D (**4**).

The extract induced apoptosis as expected and therefore was fractionated, with subsequent purification by analytical RPHPLC. Using this strategy, not only ammonificins A (**1**) and B (**2**) but also two other compounds **3** and **4** were isolated. The chemical structures of these two newly isolated compounds (**3** and **4**) were ascertained by standard spectroscopic techniques (Figure 2).

**Figure 3:**
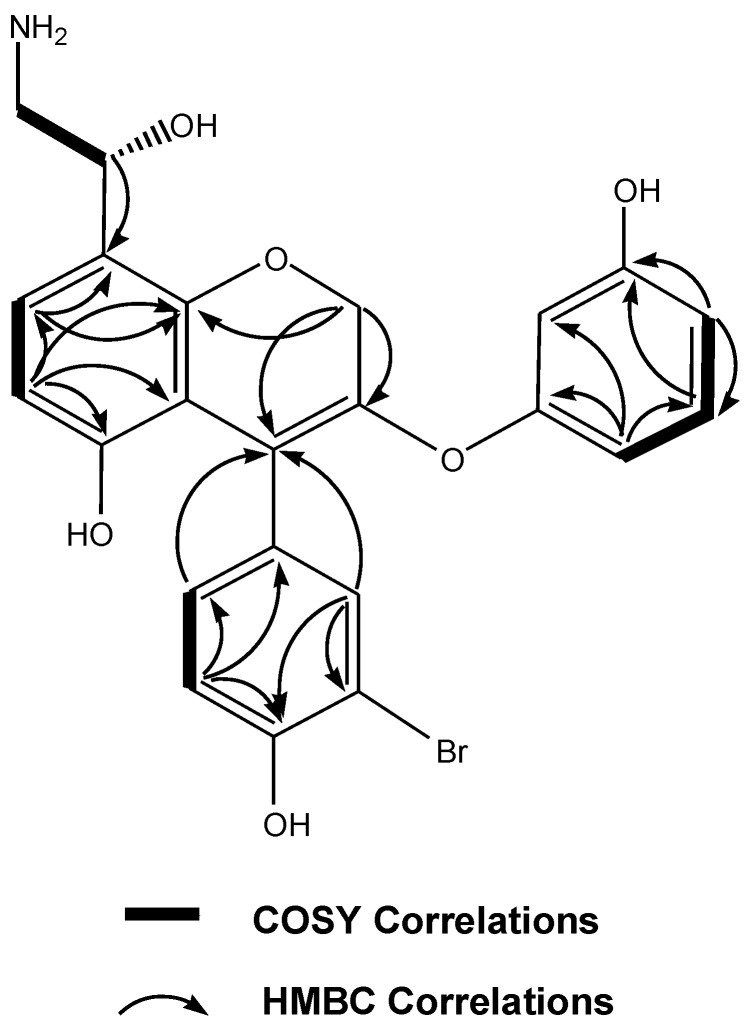
Key HMBC and selected COSY correlations for ammonificin C (**3**).

The LR ESIMS of ammonificin C (**3**) displayed ion clusters at *m*/*z* 486 (100)/488 (98) indicating the presence of one bromine. Comparison of LR ESIMS of ammonificin A (**1**) and ammonificin C (**3**) revealed that they only differ with 2 mass units implying that they share a common structural feature. The molecular formula of **3** was established as C_23_H_20_BrNO_6_ on the basis of HR ESIMS [*m*/*z* 486.0550 (M + H)^+^]. This molecular formula implied that **3** has two less hydrogen atom than **1** which is consistent with the 2 mass unit difference and keeping this in mind, the strategy for all NMR interpretation for the resolution of the structure of **3** is similar to **1** as described in our previous work [8]. The ^1^H spectrum of **3** indicated clearly the presence of nine aromatic ring signals; δ_H_ 6.67 [(d, *J* = 7.9 Hz), H-6], δ_H_ 7.09 [(d, *J* = 7.9 Hz), H-7], δ_H_ 7.41 [s, H-12], δ_H_ 6.77 [(d, *J* = 7.2 Hz), H-15], δ_H_ 7.26 [(d, *J* = 7.2 Hz), H-16], δ_H_ 6.65 [s, H-18], δ_H_ 6.74 [(d, *J* = 7.8 Hz), H-20], δ_H_ 7.01 [(dd, *J* = 7.8 Hz, 7.6 Hz), H-21], and δ_H_ 6.81 [(d, *J* = 7.6 Hz), H-22]. Their corresponding methine carbons were assigned from multiplicity edited HSQC: C-6 (δ 108.0), C-7 (δ 126.8), C-12 (δ 133.1), C-15 (δ 118.4), C-16 (δ 128.8), C-18 (δ 101.4), C-20 (δ 107.3), C-21 (δ 130.5), and C-22 (δ 106.8). Analysis of HMBC and multiplicity edited HSQC data suggested the presence of eleven quaternary carbons with signals characteristic of aromatic ring systems: (δ_C_ 107.9, 110.8, 111.7, 119.7, 129.7, 141.5, 155.7, 156.9, 157.1, 157.9, and 158.9). Given the number of carbons belonging to the aromatic signals, ammonificin C (**3**) was found to possess three aromatic ring systems similar to ammonificin A [8], but has one more double bond in its structure. Furthermore, three proton signals characteristic of hydroxy groups attached to aromatic ring systems were present in the ^1^H spectrum; δ_H_ 8.48 (br s), δ_H_ 9.26 (br s), δ_H_ 9.47 (br s). Closer examination of the ^1^H-^1^H COSY along with the ^1^H NMR spectrum and by comparison with that for ammonificin A [8] clearly indicated that the two signals characteristic of a dihydropyran moiety: δ_H_ 4.35, H-4 and δ_H_ 4.98, H-3 were missing in **3** implying that the extra double bond present in **3** is located between C-3 and C-4. HMBC correlations between H-6 and C-7 (δ_C_ 126.8), C-5 (δ_C_ 155.7), C-4a (δ_C_ 107.9), H-7 and C-8 (δ_C_ 119.7), C-8a (δ_C_ 157.9), H-2a and C-8a (δ_C_ 157.9), C-4 (δ_C_ 111.7), H-2b and C-3 (δ_C_ 141.5) strongly suggested that **3** has a chromene moiety in its structure. Another interesting group resulting from the ^1^H-^1^H COSY analysis is a hydroxyethylamine moiety [9] in **3**; δ_H_ 4.70 [m, H-9], δ_H_ 3.45 [m, H-10]. Moreover, this hydroxyethylamine moiety is found to be attached to C-8 according to the HMBC correlation between H-9 and C-8 (δ_C_ 119.7). The two remaining aromatic rings were established using ^1^H-^1^H COSY and HMBC correlations (Figure 3). The ^1^H-^1^H COSY correlation between H-15 and H-16, HMBC correlations between H-15 and C-11, C-13, C-14, C-16, and HMBC correlations between H-12 and C-13, C-14, C-16 define a trisubstituted aromatic ring system. The ^1^H-^1^H COSY correlations between H-21 and H-20, H-22, and HMBC correlations between H-20 and C-19, C-22 and between H-22 and C-17, C-18, C-20 generated a disubstituted aromatic ring system. The connectivity between the chromene moiety and one of the remaining two ring systems was established by HMBC correlations: H-12 to C-4 and H-16 to C-4 which implied that the other ring system was attached to the quarternary carbon C-3 with an ether bond to the quaternary carbon C-17. The chemical shifts of the quaternary carbons belonging to the aromatic ring systems played an important role in the assignment of the regiochemistry [8]. For example, the chemical shift of the C-5 quaternary carbon (δ_C_ 155.7) indicated that hydroxy was attached whereas the shift at the C-13 quaternary carbon (δ_C_ 110.8) indicated bromine was present. Similarly, the chemical shifts at C-14 (δ_C_ 157.1) and C-19 (δ_C_ 156.9) indicated that hydroxys were attached to these positions. 

In order to elucidate the absolute configuration at C-9 three different approaches were assessed; First, chemical derivatization of the hydroxyl attached to C-9 followed by NMR analyses of the derivative product. This approach was ruled out since ammonificin C (**3**) has three different hydroxyl groups on its structure and the chemical reaction is probably not selective. Second, crystallization of **3** given the fact that brominated molecules may crystallize easily. Previous attempt to crystallize **1** with the quantity of material available was unsuccessful so was also abandoned this approach since the quantity of material available for **3** is probably not enough for the process. Third, circular dichroism analyses of **3**, this approach present the advantage of non destructive material and basically a comparison of experimental and predicted CD spectra. Circular dichroism (CD) spectrum of ammonificin C (**3**) was obtained. This experimental CD spectrum was then compared to the predicted CD spectra of all possible stereoisomers (9*R*, 9*S*). 

**Figure 4:**
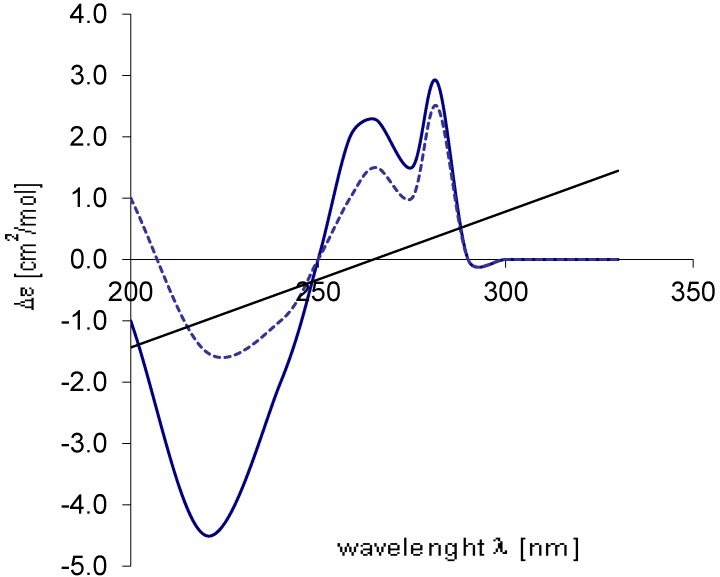
Comparison of the experimental CD spectrum (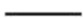) of **3** with the spectrum calculated (...........) for 9*R*.

These two probable stereoisomers were submitted to geometry optimization by the DFT (BLYP/6-31G*) approach [8,10]. For each minimized geometry a single CD spectrum was calculated using the TDDFT approach (B3LYP/TZVP) [8,10]. The overall CD spectra thus obtained were subsequently UV-corrected and compared with the experimental one of **3**. An excellent agreement between the CD curve calculated for 9*R* and the experimental was found (Figure 4).This indicated that **3** has the 9*R* configuration and the structure of **3** is established as shown.

The LR ESIMS of ammonificin D (**4**) displayed ion clusters at *m*/*z* 548 (51)/550 (100)/552 (48) indicating the presence of two bromines similar to that of found in ammonificin B (**2**). The molecular formula of **4** was established as C_23_H_19_Br_2_NO_5_ on the basis of HR ESIMS [*m*/*z* 547.9707 (M + H)^+^]. The molecular formula of **4** showed that it has two less hydrogen atome compared to **2** and one more bromine atom and one less hydrogen and oxygen atom compared to **3**. 

**Figure 5:**
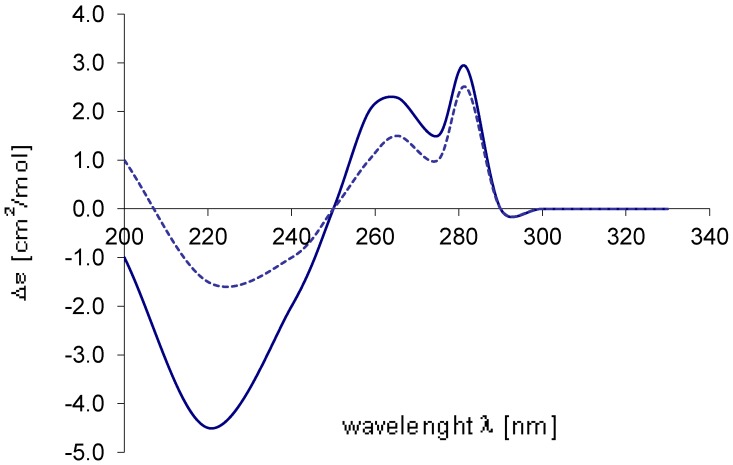
Comparison of the experimental CD spectrum (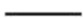) of **4** with the spectrum calculated (...........) for 9*R*.

The strong similarity of its ^1^H NMR spectrum to that of ammonificins B (**2**) and C (**3**) revealed that they share the same general structural features. Furthermore, only two proton signals characteristic of hydroxy groups attached to the aromatic ring system were present in the ^1^H spectrum of **4** (δ_H_ 9.26 (br s), δ_H_ 9.47 (br s)), suggesting that one hydroxy group was replaced by one bromine atom. HMBC correlations between H-16 and C-14 and also between H-12 and C-14 confirmed this suggestion. From the above analyses, it was concluded that the structure of **4** is first, similar to that of **3** except that the hydroxy group attached to C-14 was replaced by one bromine atom and second, similar to that of **2** except that **4** has an extra double bond between C-3 and C-4. The absolute configurations at C-9 of ammonificin D (**4**) were ascertained by the same methods as described above (Figure 5). 

Although chromene derivatives are known structures, the co-occurrence of hydroxyethylamine and phenol or brominated phenol in **3** or **4** with chromene is unique and never encounter from shallow water organism.

## 3. Experimental Section

### 3.1. General Experimental Procedures

Optical rotations were measured on a JASCO P 1010 polarimeter. UV and FT-IR spectra were obtained employing Hewlett-Packard 8452A and Nicolet 510 instruments, respectively. CD spectra were acquired on JASCO J-720 spectropolarimeter. All NMR spectra were recorded on a Bruker Avance DRX400 spectrometer, Varian-400 instrument (400 MHz) and Bruker Avance DRX600 spectrometer (600 MHz). Spectra were referenced to residual solvent signal with resonances at δ_H/C_ 2.50/39.51 (DMSO-*d*_6_). ESI MS data were acquired on a Waters Micromass LCT Classic mass spectrometer and Varian 500-MS LC Ion Trap. HPLC separations were performed using Waters 510 HPLC pumps, a Waters 717 plus autosampler, and Waters 996 photodiode array detector. All solvents were purchased as HPLC grade.

### 3.2. Extraction and Isolation Procedures

#### 3.2.1. Collection of Organism [4]

Fragments of several active black smoker chimneys were collected from the East Pacific Rise (9°50′ N, 104°189′ W) at a depth of 2500 m, during two cruises aboard RV Atlantis (November 1999 and April 2000). Samples were collected by using the manipulator of the deep-submergence vehicle Alvin and stored in boxes on the submersible’s working platform for the rest of the dive. On the surface, samples were transferred promptly to the ship’s laboratory and subsamples were placed in stoppered tubes, reduced with a 5% solution of Na_2_S and stored at 4 °C. Portions of the subsamples were used immediately for shipboard inocula by injecting 1 mL slurry (obtained by resuspension of about 1 g chimney rock in 1 mL anaerobic, sterile, artificial sea water) into 10 mL culture medium.

#### 3.2.2. Culture [4]

Isolate *Thermovibrio ammonificans* was grown routinely in modified SME medium [11], which contained (L^−1^): NaCl, 20.0 g; MgSO_4_·7H_2_O, 3.5 g; MgCl_2_·6H_2_O, 2.75 g; KCl, 0.325 g; KNO_3_, 1.0 g; NaBr, 50.0 mg; H_3_BO_3_, 15.0 mg; SrCl_2_·6H_2_O, 7.5 mg; (NH_4_)_2_SO_4_, 10.0 mg; KI, 0.05 mg; Na_2_WO_2_·2H_2_O, 0.1 mg; CaCl_2_·2H_2_O, 0.75 g; KH_2_PO_4_, 0.5 g; NiCl_2_·6H_2_O, 2.0 mg; resazurin, 1.0 mg; trace element solution, 10 mL [12]. After solubilization, the medium was heated to boiling point and then cooled under a stream of N_2_ for 30 min. Na_2_S·9H_2_O (0.5 g L^−1^) was added to reduce the medium and the pH was adjusted to 5.5 with H_2_SO_4_. The medium was then aliquoted (10 mL portions) into tightly stoppered tubes (Bellco Glass) and autoclaved (200 kPa, 20 min, 121 °C). Prior to inoculation, the medium was supplemented aseptically with 0.25 mL MES buffer (20%, w/v; pH 5.5), 0.1 mL KNO_3_ (10%, w/v) and 0.04 mL Na_2_S·9H_2_O (3%, w/v; pH 7.0); it was pressurized with H_2_/CO_2_ (80:20; 200 kPa). Cultures were incubated at 75 °C. Stocks of strain HB-1T for long-term storage were prepared by supplementing 1 mL culture with 50 μL DMSO (Fisher Scientific, Pittsburgh, USA) and were stored at −80 °C. Growth of strain *Thermovibrio ammonificans* was determined by direct counts of acridine orange-stained cells by epifluorescence microscopy, using an ocular grid. For the purpose of this study, bacterial cells were harvested from a total of 5 L of bacterial culture.

The material (40 g) was extracted three times with MeOH to give a polar crude organic extract (550 mg). A portion of this extract (20 mg) was tested for apoptosis induction. The crude organic extract was found active and subjected to fractionation using solid phase extraction cartridge (normal phase silica) to give four fractions F1 to F4 using hexane, Hex-EtOH, EtOH and MeOH as an increasingly hydrophilic solvent system series. The fractions eluting with MeOH (F4) had apoptosis induction activity. This fraction was further chromatographed by analytical RP HPLC (Phenomenex luna C18, 250 × 4.60 mm; using isocratic elution with 50% MeOH and 50% H_2_O, flow rate 1 mL/min) to yield 1.7 mg of **1 **(*t*_R_ = 6.1 min) and 1.6 mg of **2 **(*t*_R_ = 6.7 min) from F4.

Ammonificin C (**3**): [α]^24^_D_ −45 (*c* 0.8, MeOH); UV (EtOH) λ_max_ (log ε ) 267 (2.90), 285 (3.68), 305 (3.70); CD (EtOH) see Figure 3; IR ν_max_ (neat) 3350, 2950, 1620, 1460, 1380, 1230, 1160, 1120, 1090, 1020, 805 cm^−1^; ^1^H NMR and ^13^C NMR, see Table 1; HR ESIMS [*m*/*z* 486.0550 (M + H)^+^ (calcd for C_23_H_21_BrNO_6_, 486.0552)].

Ammonificin D (**4**): [α]^24^_D_ −45 (*c* 0.8, MeOH); UV (EtOH) λ_max_ (log ε) 267 (2.90), 285 (3.68), 305 (3.65); CD (EtOH) see Figure 4; IR ν_max_ (neat) 3350, 2950, 1620, 1460, 1380, 1230, 1160, 1120, 1090, 1020, 805 cm^−1^; ^1^H NMR and ^13^C NMR, see Table 2; HR ESIMS [*m*/*z* 547.9707 (M + H)^+^ (calcd for C_23_H_20_Br_2_NO_5_, 547.9708)].

**Table 1:** NMR Spectroscopic Data of Ammonificin C (**3**) (^1^H 400 MHz, ^13^C 100 MHz, DMSO-*d_6_*).

Position	δ_C_ type	δ_H_ mult. ( *J* in Hz)	HMBC ^a^
2a	76.2, CH_2_	4.41, d (6.1)	8 ^a^, 4
2b		4.10, d (6.1)	3
3	141.5, qC		
4	111.7, qC		
4a	107.9, qC		
5	155.7, qC		
6	108.0, CH	6.67, d (7.9)	4 ^a^, 5, 7, 8 ^a^
7	126.8, CH	7.09, d (7.9)	5, 8, 8 ^a^
8	119.7, qC		
8a	157.9, qC		
9	69.6, CH	4.70, m	7, 8
10	49.2, CH_2_	3.45, m	8, 9
11	129.7, qC		
12	133.1, CH	7.30, s	4,11, 13, 14, 16
13	110.8, qC		
14	157.1, qC		
15	118.4, CH	6.77, d (7.2)	11, 13, 14, 16
16	128.8, CH	7.26, d (7.2)	4,11, 12, 14, 15
17	158.9, qC		
18	101.4, CH	6.65, s	17, 19, 20, 22
19	156.9, qC		
20	107.3, CH	6.74, d (7.8)	18, 19, 21, 22
21	130.5, CH	7.01, dd (7.6,7.8)	17, 19, 20, 22
22	106.8, CH	6.81, d (7.6)	17, 18, 20, 21
OH on C-5		9.26, br s	
OH on C-14		8.48, br s	
OH on C-19		9.47, br s	

^a^ HMBC correlations, optimized for 8 or 6.5 Hz, are from proton(s) stated to the indicated carbon.

**Table 2:** NMR Spectroscopic Data of Ammonificin D (**4**) (^1^H600 MHz, ^13^C 150 MHz, DMSO-*d_6_*).

Position	δ_C_ type	δ_H_ mult. ( *J* in Hz)	HMBC ^a^
2a	76.2, CH_2_	4.41, d (6.1)	8 ^a^, 4
2b		4.10, d (6.1)	3
3	141.5, qC		
4	111.7, qC		
4a	107.91, qC		
5	155.7, qC		
6	108.0, CH	6.67, d (7.9)	4 ^a^, 5, 7, 8 ^a^
7	126.8, CH	7.09, d (7.9)	5, 8, 8 ^a^
8	119.7, qC		
8a	157.9, qC		
9	69.6, CH	4.65, t (4.3)	7, 8
10	49.2, CH_2_	3.45, m	8, 9
11	136.1, qC		
12	133.9, CH	7.26, s	4, 11, 13, 14, 16
13	126.9, qC		
14	123.9, qC		
15	132.5, CH	7.28, d (7.2)	11, 13, 14, 16
16	129.6, CH	7.24, d (7.2)	4, 11, 12, 14, 15
17	158.9, qC		
18	101.4, CH	6.65, s	17, 19, 20, 22
19	156.9, qC		
20	107.3, CH	6.74, d (7.8)	18, 19, 21, 22
21	130.5, CH	7.01, dd (7.6,7.8)	17, 19, 20, 22
22	106.8, CH	6.81, d (7.6)	17, 18, 20, 21
OH on C-5		9.26, br s	
OH on C-19		9.47, br s	

^a^ HMBC correlations, optimized for 8 or 6.5 Hz, are from proton(s) stated to the indicated carbon.

### 3.3. Computational Methods

Geometry optimization, UV and CD computations were undertaken using TDDFT with the B3LYP hybrid functional and a TZVP basis set, as included in the TURBOMOLE suite of programs with TmoleX a graphical user interface to the turbomole quantum chemistry program package [10]. The corresponding oscillator and rotatory strengths thus obtained were summed and energetically weighted, following the Boltzmann statistics. Finally, the overall UV and CD spectra were simulated as sums of Gaussian functions centered at the wavelengths of the respective electronic transitions and multiplied by the corresponding oscillator or rotatory strengths, transformed into absorption and Δε values, respectively [13,14,15,16].

### 3.4. Biological Evaluation—Apoptosis Induction

Apoptosis induction in the presence of compounds **3** and **4** was carried out as described in Andrianasolo *et al.* 2007 using the ApopScreen assay [5,6,17,18,19,20]. In this assay viability of treated W2 (apoptosis competent) and D3 (apoptosis defective) [21] cells is measured using a modification of the MTT assay [22]. For this study, viability was measured at 0 and 48 h and differential growth was calculated in the presence of the test compounds, Staurosporine (an apoptosis inducer) as positive control, and DMSO as a negative control.

## 4. Conclusions

The isolated compounds were evaluated in the apoptosis induction assay and ammonificins C (**3**) and D (**4**) induce apoptosis at 2 μM and 3 μM respectively (the control, staurosporine, a known apoptosis inducer at 0.1 μM). The original extract showed interesting activity, and we are able to isolate and correlate the pure compounds that are responsible for the activity. In our previous investigation [8], we noticed that the minor components that could not be removed during the purification process probably have a similar structure and polarity to ammonificins A and B. We have now demonstrated that these minor components are indeed ammonificins C (**3**) and D (**4**) which are the compounds that correlate to the activity of the original extract. The new double bond conjugated with the aromatic ring present in the structures of **3** and **4** may contribute significantly to their activity [23]. Given the structural feature of these compounds, other bioactivities might be targeted such as necrosis which is an alternative form of programmed cell death, antibacterial, antiviral and anti-trypanosomal. The compounds described herein represent new chemical structures and may have important potential in future drug discovery and development efforts.
